# Exposure to silica and systemic sclerosis: A retrospective cohort study based on the Canadian Scleroderma Research Group

**DOI:** 10.3389/fmed.2022.984907

**Published:** 2022-09-29

**Authors:** Anastasiya Muntyanu, Raymond Milan, Elham Rahme, Avery LaChance, Lydia Ouchene, Maxime Cormier, Ivan V. Litvinov, Marie Hudson, Murray Baron, Elena Netchiporouk, the Canadian Scleroderma Research Group, M. Baron

**Affiliations:** Montreal, Quebec; Montreal, Quebec; Montreal, Quebec; London, Ontario; Hamilton, Ontario; Hamilton, Ontario; Sherbrooke, Quebec; Halifax, Nova Scotia; Mexico City, Mexico; Ottawa, Ontario; Newmarket, Ontario; Quebec, Quebec; Quebec, Quebec; Winnipeg, Manitoba; Edmonton, Alberta; Calgary, Alberta; Moncton, New Brunswick; Ottawa, Ontario; Department of Medicine, Cumming School of Medicine, Calgary, Alberta.; ^1^Division of Dermatology, Department of Medicine, McGill University Health Centre, Montreal, QC, Canada; ^2^Division of Experimental Medicine, Department of Medicine, McGill University, Montreal, QC, Canada; ^3^Centre for Outcomes Research and Evaluation, Research Institute of the McGill University Health Centre, Montreal, QC, Canada; ^4^Division of Clinical Epidemiology, Department of Medicine, McGill University, Montreal, QC, Canada; ^5^Department of Dermatology, Brigham and Women’s Hospital, Harvard Medical School, Boston, MA, United States; ^6^Division of Respiratory Medicine, Department of Medicine, McGill University Health Centre, Montreal, QC, Canada; ^7^Division of Dermatology, University of Ottawa, Ottawa, ON, Canada; ^8^Lady Davis Institute, Jewish General Hospital, McGill University, Montréal, QC, Canada; ^9^Division of Rheumatology, Department of Medicine, Jewish General Hospital, Montreal, QC, Canada

**Keywords:** systemic sclerosis, silica, environmental triggers, occupation, mortality, gastrointestinal disease, interstitial lung disease, scleroderma

## Abstract

**Introduction:**

Systemic sclerosis (SSc) is thought to be induced by an environmental trigger in genetically predisposed individuals. This study assessed the demographic and clinical characteristics and disease severity of silica exposed SSc patients.

**Methods:**

Data was obtained from the Canadian Scleroderma Research Group (CSRG) cohort, containing 1,439 patients (2004–2019). Univariate and multivariate logistic regression analyses were performed, to determine the phenotype and severity of silica-exposed SSc patients. Mortality was assessed using Cox Survival Regression and Kaplan-Meier analyses.

**Results:**

Among 1,439 patients (86.7% females), 95 patients reported exposure to silica. Those exposed were younger, of male sex and with more severe disease. Sex differences were observed where male patients exposed to silica were more likely to be Caucasian and smokers whereas female patients were younger at SSc diagnosis compared to unexposed. Multivariate regression, controlled for multiple confounders, showed that silica exposure was associated with a younger age at diagnosis and worse disease severity and mortality.

**Conclusion:**

Exposure to silica was reported in ∼7% of CSRG cohort and ∼20% of male patients and was associated with a worse prognosis in terms of age of diagnosis, organ involvement and mortality. Hence, screening for silica exposure among higher risk individuals may be beneficial and these patients may require closer monitoring for systemic disease.

## Introduction

Systemic sclerosis (SSc) is a chronic, fibrosing systemic autoimmune rheumatic disease (1). Most commonly affected organs are the skin, gastrointestinal (GI) tract, and lungs, which have the most contact to the outside world, and their involvement leads to significant morbidity and mortality (2). The prevalence of SSc in Canada in 2003 was estimated to be 74.4/100,000 females and 13.3/100,000 males (2). While SSc is more common in females, the prognosis has been consistently shown to be worse in male patients including more diffuse cutaneous SSc (dcSSc), more interstitial lung disease (ILD) and higher mortality (3, 4). It is not known whether this different SSc phenotype and prognosis seen in males is mediated by biological/hormonal influences or whether exposure/occupation related factors in males contribute to the process. As disease-modifying treatment options are limited, determining triggers and elucidating preventive strategies is of significant importance (3).

The pathogenesis of SSc, while not fully understood, is believed to be induced by environmental triggers in genetically predisposed hosts (3, 4). The nature of such triggers and factors accounting for disease severity/prognosis remain poorly understood. A recent review highlighted the environmental factors studied to date for association with SSc (5). The strongest evidence was observed for environmental or occupational exposure to silica and organic solvents. Specifically, a meta-analysis of cohort studies focusing on workers (often male) exposed to silica demonstrated an 18-fold increased incidence of SSc (5).

While there is evidence to suggest that occupational exposure to silica may be associated with an increased risk of SSc and a more severe phenotype, the proportion of SSc patients with history of occupational or other exposure to silica in North America remains to be clarified. It is unclear whether certain patient characteristics should prompt assessment for prior silica exposure and whether these patients have a more severe SSc than unexposed SSc patients and may require a different clinical/screening approach for comorbidities and complications. Hence, using the Canadian Scleroderma Research Group (CSRG) (6), we aimed to assess the frequency of occupational exposure to silica among Canadian SSc patients, define the demographic and clinical characteristics of SSc patients exposed to silica, and study whether occupational exposure to silica confers a worse disease severity and mortality.

## Materials and methods

### Study population

The CSRG is the largest multi-center registry of Canadian SSc patients, extensively described elsewhere (7–10). Patients with a diagnosis of SSc followed in one of the 15 rheumatology centers (across Canada and Mexico), who accepted to participate in the registry, were prospectively recruited between 2004 and 2019. Detailed demographic, clinical, laboratory and imaging data were collected at study enrollment (first visit) and at subsequent visits (usually annually) thereafter, for up to 15 study visits. SSc diagnosis was verified by an experienced rheumatologist and over 98% of the patients met the 2013 ACR/EULAR classification criteria for SSc (11). Ethics approval for this study was obtained at the Jewish General Hospital, Montreal, Canada and at all participating CSRG study sites.

### Design

This retrospective cohort study included SSc patients with ≥ 1 registry visit between January 2004 and September 2019. Patients were asked to complete a detailed questionnaire at inception into the cohort which included yes/no questions regarding certain occupational exposures. Patients were categorized into silica exposed vs. silica unexposed based on their response. The following was indicated on the form “Please check the box if you have ever worked in environments that commonly involve the following substances or if you have ever been exposed to the medications or other exposures mentioned below. If you are not sure or would like to comment, please use the space provided.” A patient was considered to be exposed to silica if they answered yes to any of the following exposures: silica dusts, hard rock mining, and/or coal mining. Patients also had an optional field to enter their current occupation title.

### Socio-demographic and clinical characteristics

The following variables were extracted from the first patient’s visit for all exposure groups: age, sex, ethnicity, smoking status (never or ever smoked), and disease duration (defined as the time between the onset of first non-Raynaud manifestation and recruitment date into the study). SSc subtype was reported as limited cutaneous (lcSSc) and dcSSc, defined as skin fibrosis involving the proximal limbs and/or trunk at any time (8). Severity of skin involvement was measured using the modified Rodnan skin score (mRSS) (score range 0–51) (8). Presence of abnormal nailfold capillaroscopy and history of finger necrosis/gangrene/amputation were recorded.

Presence and severity of internal organ involvement (cutaneous, cardiac, pulmonary, renal, and GI), SSc-specific and SSc-related antibodies and treatment were collected at baseline. Systemic organ involvement was defined as follows (ILD defined below). Pulmonary hypertension corresponds to a systolic pulmonary artery pressure of > 45 mmHg on right heart echocardiogram. SSc-specific renal involvement was defined as a history of renal crisis. Gastrointestinal (GI) involvement was defined based on the median gastrointestinal (GI)-14 score (12). Other variables recorded by recruiting physicians at the first visit included presence of inflammatory arthritis, myositis, and history of cancer. Mean Medsger disease severity scale (DSS) (13), assessing the presence and severity of 9 individual organs, was also evaluated to corroborate results (14). Categories in this scale included a general domain, peripheral vascular, joint/tendon, muscle, GI, pulmonary, cardiac, renal, and the skin domain. Detailed definition and grading of Medsger DSS are explained elsewhere (14).

Antibody profiles including anticentromere (ACA), anti-topoisomerase 1 (ATA), anti-RNA polymerase III antibodies (anti-RNAP), anti-Ro52, and anti- nucleolus organizer region 90 (Nor90), anti-Ku, anti-Th/To, fibrillarin, anti-PM75, and anti-PM100 were detected by Euroline SSc profile LIA (Euroimmun GmbH, Luebeck, Germany) according to manufacturer’s instructions. Antibody against anti-U1 ribonucleoproteins (U1RNP) was assessed by addressable laser bead immunoassays (ALBIA) (QUANTA PlexTM SLE8, INOVA Diagnostics, Inc.). All measurements were obtained from the initial registry visit. Antibodies were reported as negative or weak positive (considered absent) and moderate or strong positive (considered present) based on the accepted lab cut off point and numerical values were not available. Antibodies with nucleolar patterns were considered to be fibrillarin, anti-Th/To, Anti RNAP, PM75 and PM100 (15).

Medication history, including mycophenolate mofetil (MMF) or cyclophosphamide (CYC), was recorded by recruiting physicians as past use, current use, or never used. This was dichotomized for statistical analysis into exposed (past or current use) and never exposed.

### Disease severity definition(s)

The following disease severity outcomes were considered: dcSSc phenotype, SSc-specific antibodies, younger age at SSc diagnosis, worse GI disease (GI-14 score) and higher risk and worse ILD. ILD was defined as present if a High Resolution Computerized Tomography (HRCT) of the lungs was interpreted by an experienced radiologist as showing ILD or chest x-ray findings of increased interstitial markings (not due to congestive heart failure) or fibrosis, and/or if a study physician reported findings indicative of ILD on physical examination based on a previously published decision rule (16). Patients with ILD were stratified into Forced Vital Capacity (FVC) of ≥ 70% for mild disease and < 70% for moderate-to-severe based on their spirometry findings on the first visit.

### Mortality

Mortality data was collected during annual visits using a standardized death case report form (17). Follow up was started at date of first registry visit and end of follow up was considered when mortality occurred. Patients were censored at the last available registry visit if they were lost to follow up and no mortality data was recorded.

### Statistical analysis

Baseline patient characteristics were compared across the two groups (silica exposure vs. no silica exposure) using Chi-square or Fisher’s exact test for categorical variables and ANOVA or Kruskal-Wallis test for continuous variables. Univariate and multivariate logistic regression was used to predict patients’ characteristics associated with silica exposure, where silica exposure was considered as the outcome.

Additional univariate logistic regression models, where silica exposure was considered to be a predictor, were used for categorical variables and linear regression for the continuous variable (i.e., GI-14) to determine whether silica exposure was associated with SSc severity (as defined above). The multivariate model was adjusted for possible confounders.

Cox regression analysis for mortality was performed adjusting for age, sex, smoking, and disease duration. R Studio (version 1.4.1106) and SAS studio software was used to conduct all statistical analyses.

### Subgroup analyses

As silica exposure is more common in males, separate analyses for silica exposure were performed by sex. Data on gender was not available.

## Results

### Patient characteristics

In total, 1,439 patients were included in this study, 86.7% were females with mean age at SSc diagnosis of 46.5 ± 13.7 years. Average disease duration at baseline was 9.83 ± 9.23 years.

Ninety-five patients (6.6%) reported exposure to silica with female-male ratio of ∼1:1 among exposed vs. 8:1 among unexposed patients (6.5:1 in the entire CSRG cohort). Specifically, 22.4% of CSRG males vs. 4.2% females were exposed to silica (*p* < 0.0001) (Table 1).

**TABLE 1:** Baseline patient characteristics.

Variables	Exposure to silica (*N* = 95)	No exposure to silica (*N* = 1,344)	*P*
**Demographics**			
Age ≥ 50 years, *N* (%)	25 (27.5)	546 (41.5)	**0.016**
Male sex, *N* (%)	43 (45.3)	149 (11.1)	**<0.001**
Caucasian, *N* (%)	88 (92.6)	1,204 (89.7)	0.626
Disease duration ≥ 5 years, *N* (%)	47 (51.6)	773 (58.9)	0.207
Smoking, *N* (%)	67 (70.5)	786 (58.7)	0.065
**Clinical characteristics**			
Diffuse disease, *N* (%)	48 (51.6)	470 (35.3)	**0.003**
Treatment with CYC or MMF, *N* (%)	14 (14.9)	110 (8.3)	0.082
ILD, *N* (%)	36 (38.3)	393 (30.0)	0.157
FVC < 70, *N* (%) (*n* = 1,244)	18 (21.7)	130 (11.2)	**0.016**
Pulmonary arterial hypertension, *N* (%) (*n* = 909)	8 (15.4)	140 (16.3)	0.208
Rodnan score, median (IQR)	10.00 [4.00, 17.50]	6.00 [2.00, 14.00]	**0.011**
Digital ulcer/pitting scars, *N* (%)	45 (47.9)	556 (41.8)	0.516
Necrosis/gangrene/amputation, *N* (%)	32 (34.0)	473 (35.6)	0.957
Nailfold capillaroscopy, *N* (%)	67 (71.3)	1,017 (76.5)	0.513
GI_14, Median (IQR) (*n* = 1,343)	4.00 [2.00, 7.00]	3.00 [1.00, 6.00]	**0.014**
Joint impairment, *N* (%)	23 (24.5)	377 (28.3)	0.721
History of renal crisis, *N* (%)	7 (7.4)	48 (3.6)	0.174
Cancer, *N* (%)	8 (8.5)	108 (8.1)	0.991
**Antibody profile**			
ACA antibody, *N* (%) (*n* = 1,244)	24 (28.3)	458 (39.5)	0.075
ATA antibody, *N* (%) (*n* = 1,244)	18 (21.8)	172 (14.8)	0.189
U1RNP, *N* (%) (*n* = 1,276)	6 (7.0)	63 (5.3)	0.758
Ro52, *N* (%) (*n* = 1,244)	22 (25.8)	309 (26.7)	0.664
Ku, *N* (%) (*n* = 1,244)	0 (0.0)	9 (0.7)	0.477
Nor90, *N* (%) (*n* = 1,244)	1 (1.1)	26 (2.2)	0.54
Nucleolar antibodies, *N* (%) (*n* = 1,243)	18 (21.4)	232 (20.0)	0.795
**Medsger severity scores**			
Medsger—general, mean (*SD*)	1.06 (1.37)	0.89 (1.18)	0.172
Medsger—peripheral vascular, Mean (*SD*) (*n* = 1,156)	1.88 (1.21)	1.63 (1.24)	0.097
Medsger—skin, mean (*SD*)	1.36 (0.77)	1.21 (0.70)	0.05
Medsger—joint/tendon, Mean (*SD*) (*n* = 1,095)	0.96 (1.32)	0.68 (1.18)	0.055
Medsger—muscle, mean (*SD*)	0.33 (0.90)	0.23 (0.72)	0.185
Medsger—GI tract, mean (*SD*)	2.07 (0.85)	1.91 (0.78)	**0.049**
Medsger—lung, mean (*SD*)	1.57 (1.21)	1.30 (1.11)	**0.02**
Medsger—heart, mean (*SD*)	0.56 (1.17)	0.46 (0.95)	0.354
Medsger—kidney, mean (*SD*) (*n* = 1,266)	0.16 (0.65)	0.11 (0.61)	0.473

IQR, interquartile range; SD, standard deviation; CYC, cyclophosphamide; MMF, mycophenolate mofetil; ACA, anticentromere antibody; ATA, anti-topoisomerase I antibody; U1RNP, anti-U1 Ribonucleoproteins antibody; ILD, interstitial lung disease; FVC, forced vital capacity; Unless a different denominator (n) is indicated, the missing number for remaining variables was < 5%.

Chi-square or fisher exact test for categorical variable. ANOVA or Kruskal-Wallis test for continuous variables.

N provided where > 5% of data was missing.

Bold indicates statistically significant values.

Baseline patient characteristics (Table 1) showed that SSc patients exposed to silica were significantly more likely to be younger at diagnosis (median age 44.9 vs. 47.2 years old; *p* = 0.016), males (45.3 vs. 11.1%; *p* < 0.001), have a dcSSc phenotype (51.6% vs. 35.3%; *p* = 0.003), more severe ILD (with higher proportion of low FVC (< 70%) 21.7% vs. 11.2%; *p* = 0.016; higher Medsger score for lung disease 1.57 vs. 1.30; *p* = 0.02), worse skin fibrosis based on mRSS, 10 vs. 6; *p* = 0.011), and worse GI disease (median GI-14 score, 4 vs. 3; *p* = 0.014 and Medsger GI score 2.07 vs. 1.91; *p* = 0.049) compared to the non-exposed group. Furthermore, consistent with dcSSc phenotype, silica-exposed patients had higher ATA positivity (21.8% vs. 14.8%), lower ACA positivity (28.3% vs. 39.5%), higher prevalence of ILD (38.3% vs. 30.0%), and were more likely to be treated with cyclophosphamide (CYC) and/or MMF (14.9% vs. 8.3%), albeit statistical significance was not reached.

Results of the univariate logistic regression were similar. Silica-exposed patients were younger at diagnosis (OR 0.53; 95% CI: 0.33–0.84), males (OR 6.63; 95% CI: 4.27–10.28), and smokers (OR 1.69; 95% CI: 1.08–2.69) with worse disease phenotype, notably dcSSc (OR 1.96; 95% CI: 1.28–2.99), treatment with CYC and/or MMF (OR 1.95; 95%CI: 1.03–3.45), lower ACA positivity (OR 0.60; 95% CI: 0.36–0.97), more severe ILD (FVC < 70% predicted, OR 2.20; 95% CI: 1.23–3.74 and higher mean lung Medsger severity score, OR 1.24; 95%CI: 1.03–1.48), worse skin fibrosis (mRSS, OR 1.03; 95%CI: 1.01–1.04), and worse GI disease (higher GI-14 and Medsger GI scores). All significant variables were considered in the multivariate model (Table 2). Co-linearity assessment between the included variables in the multivariate model did not identify high collinearity (*r* > 0.7) (data not shown). The results showed that younger age (OR 0.42; 95%CI: 0.22–0.75), male sex (OR 7.87; 95%CI: 4.51–13.84), severe ILD (FVC < 70%) (OR 2.08; 95%CI: 1.00–4.27) and severe GI disease (GI-14) (OR 1.11; 95%CI: 1.01–1.21) were significant demographic and clinical characteristics of silica exposed patients.

**TABLE 2:** Univariate and multivariate logistic regression model for factors associated with exposure to silica among all study sample.

	Univariate logistic regression	Multivariate logistic regression
	Silica (*N* = 95)*	Silica
Variables	OR (95% CI)	Adjusted OR (95% CI)
**Age ≥ 50 years**	**0.53 (0.33**–**0.84)**	**0.42 (0.22**–**0.75)**
**Male sex**	**6.63 (4.27**–**10.28)**	**7.87 (4.51**–**13.84)**
Caucasian	1.15 (0.71–3.51)	–
Disease duration ≥ 5 years	0.75 (0.49–1.14)	–
Treatment with CYC or MMF	**1.95 (1.03**–**3.45)**	1.11 (0.45–2.48)
Smoking	**1.69 (1.08**–**2.69)**	1.05 (0.60–1.89)
Diffuse disease	**1.96 (1.28**–**2.99)**	1.49 (0.75–2.94)
Digital ulcer/pitting scars	1.28 (0.84–1.94)	–
Necrosis/gangrene/amputation	0.94 (0.60–1.44)	–
Nailfold capillaroscopy	0.76 (0.48–1.23)	–
Pulmonary arterial hypertension	0.93 (0.40–1.92)	–
Joint impairment	0.82 (0.49–1.31)	–
History of renal crisis	2.15 (0.87–4.60)	–
Cancer	1.05 (0.46–2.10)	–
ACA antibody	**0.60 (0.36**–**0.97)**	0.91 (0.48–1.66)
ATA antibody	1.54 (0.87–2.60)	–
U1RNP	1.36 (0.51–3.00)	–
Ro52	0.96 (0.57–1.56)	–
PDGFR	–	–
Ku	–	–
Nor90	0.52 (0.03–2.49)	–
Nucleolar antibodies	1.09 (0.62–1.83)	–
ILD	1.45 (0.93–2.22)	–
FVC < 70	**2.20 (1.23**–**3.74)**	**2.08 (1.00**–**4.27)**
GI_14	**1.08 (1.02**–**1.15)**	**1.11 (1.01**–**1.21)**
Rodnan score	**1.03 (1.01**–**1.04)**	1.00 (0.96–1.03)
Medsger—General	1.12 (0.95–1.31)	–
Medsger—Peripheral Vascular	1.18 (0.97–1.43)	–
Medsger—Skin	1.31 (0.99–1.72)	–
Medsger—Joint/Tendon	1.19 (0.99–1.41)	–
Medsger—Muscle	1.17 (0.90–1.47)	–
Medsger—GI tract	**1.33 (1.01**–**1.79)**	0.99 (0.69–1.43)
Medsger—Lung	**1.24 (1.03**–**1.48)**	1.08 (0.82–1.42)
Medsger—Heart	1.10 (0.89–1.33)	–
Medsger—Kidney	1.12 (0.77–1.48)	–

* Reference group no exposure to silica.

– Defines not available/not applicable.

Bold signifies significant values; Variable definitions as above.

Multivariate regression analyses stratified by sex and adjusted for confounders (all significant variables identified in the univariate model), female patients exposed to silica were diagnosed younger (OR 0.40; 95%CI: 0.16–0.90) whereas male patients were more likely to be Caucasian (OR 12.06; 95% CI: 1.83–250.88), smokers (OR 4.70; 95% CI: 1.10–33.72), and had more severe ILD (OR 5.72; 95% CI: 1.51–24.27) (Table 3).

**TABLE 3:** Univariate and multivariate logistic regression model for factors associated with exposure to silica among male and female patients.

	Univariate logistic regression		Multivariate logistic regression	
	Males	Females	Males	Females
		
	OR (95% CI)*	OR (95% CI)*	Adjusted OR (95% CI)	Adjusted OR (95% CI)
Age ≥ 50 years	0.60 (0.29 201.21)	**0.37 (0.17 200.72)**	0.37 (0.12 201.03)	**0.40 (0.16 200.90)**
Male sex	**–**	**–**	**–**	**–**
Caucasian	**3.94 (1.10 25.15)**	1.00 (0.43 202.93)	**12.06 (1.83 250.88)**	0.95 (0.37 202.97)
Disease duration ≥ 5 years	0.87 (0.43 201.74)	0.88 (0.50 201.59)		
Treatment with CYC or MMF	1.39 (0.51 203.48)	1.89 (0.76 204.07)	0.75 (0.16 203.08)	2.20 (0.66 206.32)
Smoking	**3.22 (1.19 11.26)**	0.89 (0.51 201.57)	**4.70 (1.10 33.72)**	0.68 (0.34 201.35)
Diffuse disease	0.87 (0.44 201.72)	**2.22 (1.25 203.93)**	1.57 (0.48 205.19)	1.50 (0.60 203.67)
Digital ulcer/pitting scars	1.03 (0.52 202.06)	1.04 (0.58 201.83)	–	–
Necrosis/gangrene/amputation	0.63 (0.28 201.32)	1.27 (0.71 202.23)	–	–
Nailfold capillaroscopy	1.00 (0.48 202.19)	0.71 (0.39 201.36)	–	–
Pulmonary arterial hypertension	1.54 (0.39 205.18)	0.79 (0.23 202.08)	–	–
Joint impairment	0.50 (0.19 201.16)	1.15 (0.61 202.08)	–	–
History of renal crisis	2.03 (0.60 206.25)	1.20 (0.19 204.06)	–	–
Cancer	0.93 (0.20 203.17)	1.22 (0.41 202.86)	–	–
ACA antibody	0.95 (0.37 202.22)	0.74 (0.39 201.37)	1.95 (0.59 206.43)	0.65 (0.28 201.45)
ATA antibody	1.63 (0.67 203.76)	1.24 (0.53 202.57)	–	–
U1RNP	2.29 (0.29 14.31)	1.59 (0.47 204.10)	–	–
Ro52	0.76 (0.30 201.75)	1.19 (0.61 202.23)	–	–
PDGFR	–	–	–	–
Ku	–	–	–	–
Nor90	0.80 (0.04 205.63)	–	–	–
Nucleolar antibodies	0.72 (0.29 201.67)	1.17 (0.54 202.31)	–	–
ILD	**2.20 (1.11 204.45)**	0.74 (0.37 201.40)	2.10 (0.80 205.70)	**0.25 (0.08 200.64)**
FVC < 70	**2.88 (1.14 207.12)**	1.75 (0.74 203.67)	**5.72 (1.51 24.27)**	1.75 (0.58 205.05)
GI_14	**1.13 (1.01 201.26)**	1.10 (1.02 201.20)	1.10 (0.93 201.31)	1.10 (0.98 201.23)
Rodnan score	0.99 (0.96 201.02)	**1.03 (1.00 201.05)**	0.98 (0.92 201.04)	1.01 (0.97 201.06)
Medsger—General	1.10 (0.85 201.4)	1.06 (0.93 201.60)	–	–
Medsger—Peripheral vascular	0.98 (0.72 201.34)	1.21 (0.93 201.60)	–	–
Medsger—Skin	0.80 (0.51 201.22)	1.40 (0.94 202.02)	–	–
Medsger—Joint/Tendon	0.97 (0.73 201.27)	1.19 (0.92 201.50)	–	–
Medsger—Muscle	1.65 (0.99 202.75)	1.10 (0.75 201.48)	–	–
Medsger—GI tract	1.16 (0.79 201.75)	**1.47 (1.01 202.21)**	0.77 (0.44 201.33)	1.39 (0.81 202.36)
Medsger—Lung	**1.37 (1.02 201.86)**	1.07 (0.83 201.37)	1.03 (0.65 201.63)	1.15 (0.79 201.65)
Medsger—Heart	1.09 (0.79 201.48)	1.01 (0.72 201.33)	–	–
Medsger—Kidney	1.02 (0.68 201.61)	0.89 (0.34 201.45)	–	–

*Reference group: no exposure to silica.

Bold indicates statistically significant values.

To assess whether exposure to silica may predict a worse disease prognosis, linear and logistic regression was performed (Table 4). SSc patients with reported silica exposure were significantly more likely to be diagnosed before age 50 (OR 0.53; 95% CI: (0.33–0.84) and have a worse disease. Notably, higher risk of dcSSc phenotype (OR 1.95; 95% CI: 1.28–2.99), more severe GI disease (β 0.85; 95% CI: 0.19–1.51), and a lower likelihood of ACA antibody positivity (OR 0.60; 95% CI: 0.37–0.98) were seen. A trend toward more ILD and more severe ILD was also observed, however this was not statistically significant. Multivariate model confirmed that SSc patients with silica exposure were significantly more likely to be diagnosed with both Raynaud’s phenomenon (OR 0.48, 95% CI: 0.29–0.78) or SSc before 50 years of age (OR 0.47; 95% CI: 0.29–0.77) when adjusted for sex, ethnicity, smoking status and dcSSc disease phenotype. A strong trend for increased risk of ILD, severe ILD (OR 2.05; 95% CI: 0.96–5.36) and worse GI disease (β 0.67; 95% CI: -0.03 to 1.36) was observed when adjusting for multiple confounders including sex (Table 4). Similarly, an important trend toward dcSSc phenotype (OR 1.54; 95% CI: 0.99–2.42), higher prevalence of ATA antibodies and lower prevalence of ACA antibodies was seen after adjusting for age, sex, smoking, and ethnicity.

**TABLE 4:** Association between health outcomes and exposure to silica (yes vs. no).

	Unadjusted OR (95% CI)	Adjusted OR (95% CI)	Unadjusted β (95% CI)	Adjusted β (95% CI)
ILD (yes vs. no) (*N* = 1,371)^a^	1.44 (0.94–2.23)	1.21 (0.73–1.99)	–	–
Severe ILD* (vs. mild ILD) (*N* = 370)^b^	1.60 (0.74–3.50)	2.05 (0.96–5.36)	–	–
Diffuse disease (yes vs. no) (*N* = 1,397)^c^	**1.95 (1.28**–**2.99)**	1.54 (0.99–2.42)	–	–
GI-14 (*N* = 1,397)^d^	–	–	**0.85 (0.19**–**1.51)**	0.67 (-0.03–1.36)
ATA (yes vs. no) (*N* = 1,222)^e^	1.54 (0.89–2.66)	1.47 (0.82–2.64)	–	–
ACA (yes vs. no) (*N* = 1,222)^f^	**0.60 (0.37**–**0.98)**	0.76 (0.45–1.29)	–	–
Earlier age of onset of disease ≥ 50 years (*N* = 1,395)^g^	**0.53 (0.33**–**0.84)**	**0.47 (0.29**–**0.77)**	–	–
Age of onset of Raynauds ≥ 50 years (*N* = 1,397)^h^	**0.53 (0.33**–**0.85)**	**0.48 (0.29**–**0.78)**	–	–

^a^Model adjusted for age, sex, diffuse disease, immunosuppressive medication, disease duration, ethnicity, smoking and organic solvents.

^b^Model adjusted for age, sex, diffuse disease, immunosuppressive medication, disease duration, ethnicity, smoking and organic solvents.

^c^Model adjusted for age, sex, smoking, and ethnicity.

^d^Model adjusted for age, sex, disease duration, diffuse disease, immunosuppressive medication, smoking and organic solvents.

^e^Model adjusted for age, sex, smoking, and ethnicity.

^f^Model adjusted for age, sex, smoking, and ethnicity.

^g^Model adjusted for sex, smoking, ethnicity, and diffuse disease.

^h^Model adjusted for sex, smoking, ethnicity, and diffuse disease. *Defined as presence of ILD and FVC < 70. ILD, interstitial lung disease; ACA, anticentromere antibody; ATA, anti-topoisomerase I antibody. Bold indicates statistically significant values.

### Mortality

**FIGURE 1:**
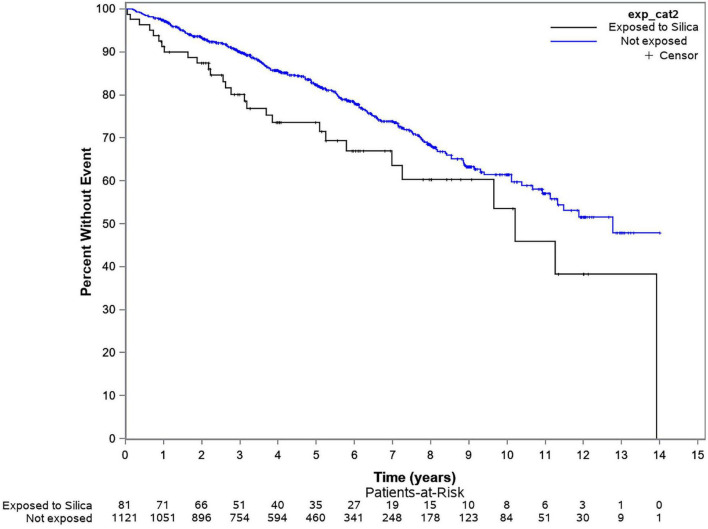
Kaplan-Meier curve evaluating mortality over time in silica exposed vs. unexposed groups.

Over the follow up period, 237 patients (of 1,439) were excluded for loss to follow up and/or missing data and 260 died (21.6%). Mortality rate of 71.4 (95% CI: 47.4–103.2) per 1,000 person-years (103.7 per 1,000 person-years in males and 50.5 per 1,000 person-years in females) was seen in silica exposed patients vs. 43.4 (95% CI: 38.0–49.4) (76.1 per 1,000 person-years in males and 39.9 per 1,000 persons-years in females) in the unexposed group (Table 5). Additionally, mortality in patients exposed to silica with disease duration of < 5 years was 86.1/1,000 person-years compared to 41.5/1,000 person years in the unexposed group. Unadjusted Kaplan Meier curve shows a significantly increased mortality rate in the silica-exposed group compared to the unexposed [Hazard Ratio (HR) 1.58, 95%CI: 1.07–2.35; *p* = 0.0217] (Figure 1). When the hazard ratio was adjusted for age, sex, smoking, and disease duration, a non-statistically significant trend for increased mortality was observed (HR 1.45, 95%CI: 0.96–2.19; *p* = 0.0911).

**TABLE 5:** Mortality case count and incidence rate per 1,000 person years.

	Number of deaths	Person-years	MR per 1,000 person-year (95% CI)
Exposure to silica (*N* = 81)	28	391	71.4 (47.4–103.2)
No exposure to silica (*N* = 1,121)	232	5,340	43.4 (38.0–49.4)
**Male patients (*N* = 162)**			
Exposure to silica (*N* = 38)	16	154	103.7 (59.3–168.4)
No exposure to silica (*N* = 124)	40	527	76.1 (54.3–103.6)
**Female patients (*N* = 1,040)**			
Exposure to silica (*N* = 43)	12	237	50.5 (26.1–88.2)
No exposure to silica (*N* = 997)	192	4,813	39.9 (34.4–45.9)
**Young patients < 50 years (*N* = 712)**			
Exposure to silica (*N* = 59)	14	305	45.9 (25.1–77.0)
No exposure to silica (*N* = 653)	110	3,211	34.2 (28.1–41.3)
**Older patients ≥ 50 years (*N* = 490)**			
Exposure to silica (*N* = 22)	14	87	161.2 (88.1–270.3)
No exposure to silica (*N* = 468)	122	2,129	57.4 (47.6–68.4)
**Disease duration < 5 year (*N* = 493)**			
Exposure to silica (*N* = 39)	13	151	86.1 (45.8–147.3)
Not exposure to silica (*N* = 454)	85	2,046	41.5 (33.2–51.4)
**Disease duration ≥ 5 year (*N* = 709)**			
Exposure to silica (*N* = 42)	15	241	62.2 (34.8–102.7)
Not exposure to silica (*N* = 667)	147	3,294	44.6 (37.7–52.4)

The whole study sample contained 1,202 patients. MR, mortality rate.

## Discussion

Occupational exposure to silica has been strongly correlated with a higher risk of developing SSc and possibly confers a worse disease phenotype, however prior studies were limited by patient number and hence additional research were needed (5). Occupations with highest risk of exposure are reviewed elsewhere (5), but these include coremaker, bench molder, mineral-crushing machine operator, stone and gem cutter and finisher, concrete-mixer operator, and miners (5). Not surprisingly these occupations commonly employ male patients and males have been consistently shown to exhibit a worse SSc-related prognosis (3).

We showed that 6.6% of the CSRG participants reported exposure to silica and higher chance of reporting silica-exposure was associated with younger age (at diagnosis), male sex and more severe disease phenotype. These findings are aligned with previous reports where Marie et al. also showed that the 18 SSc patients exposed to silica vs. 82 patients not exposed to either silica nor organic solvents in their study were more often males with severe disease (18). Our results are also aligned with a recent Australian SSc cohort study, which also found that patients exposed to silica were more likely to be male, smokers with worse disease features such as dcSSc, ILD, lower frequency of ACA (3). One study reported the dose dependent relationship between silica exposure and SSc although this could not be assessed in our cohort. The overall risk was found to increase with cumulative exposure from the time of entering the workforce for males [Incidence Rate Ratio (IRR) 1.07 (1.05–1.09) per 50 mg/m^3^ -years] and females [IRR 1.04 (0.99–1.10) per 50 mg/m^3^ -years] (19).

We found that exposure to silica in SSc patients confers a twofold increased risk of being diagnosed with Raynaud’s phenomenon and SSc before age 50, despite adjusting for multiple confounders, including sex. Furthermore, silica exposure increased the risk of dcSSc and ATA positivity by almost 50% and increased the risk of severe ILD by twofold, with a confidence interval near statistical significance despite adjusting for multiple confounders. Previous literature supports the association between ILD, dcSSc phenotype and silica exposure (5, 20, 21). As expected, ACA positivity was lower in silica exposed patients as this antibody profile is typically associated with lcSSc phenotype and has been consistently shown to be protective against ILD and SSc related mortality in both lcSSc and dcSSc.

A robust trend toward worse GI disease was seen in patients exposed to silica in our study. While previous studies have shown the association between occupational silica exposure and gastric cancer, GI symptoms secondary to silica exposure in SSc patients have not yet been reported. Silica can come into contact with the GI tract as a result of ingestion following clearance from the lungs (22, 23) and can lead to chronic local injury and inflammation in the GI tract (24). Thus, this area warrants further evaluation and assessment.

Sex is an important determinant of SSc prognosis. In our study, almost a quarter of male SSc patients reported silica exposure as opposed to only 4.2% of female SSc patients. This is also aligned with the Australian SSc cohort where 7.5% of SSc patients and 31.6% of male SSc patients reported silica exposure (3). These rates of silica exposure are much higher than rates expected in general population where ∼1.1% of working Canadians may be exposed to silica in the workplace (25). The profile of SSc patients exposed to silica differed by sex. Male patients were more likely to be younger, Caucasian and smokers whereas female SSc patients exposed to silica were more likely to be younger at diagnosis compared to silica unexposed group. Despite adjusting for sex and multiple other covariates, we showed that silica exposure was associated with adverse outcomes.

Almost 35% of silica exposed and ∼20% of unexposed SSc patients died over 14-year follow up with a mortality rate of 71.4 per 1,000 person-years in silica exposed vs. 43.4 in unexposed patients. As expected, mortality rate was higher for males and older patients. While significance was lost after adjustment, we believe the strong trend toward excess mortality needs further research.

Our study has several limitations. Missing data for individual variables usually ranged from 0 to 5% (Table 2). The exposure to silica was based on self-report and coded as Yes/No. Timing, duration, and intensity of exposure were not available, although this is similar to other studies in the literature. Hence, it was not possible to evaluate dose dependent response and development/severity of SSc. Additionally, exposure misclassification and recall bias are possible. However, we believe that both under- and overreporting of silica exposure are more likely to bias toward not finding any association. Few patients reported their occupation and industry type, which was used to verify likelihood of exposure to silica. However, different industry types and occupations may lead to low vs. high risk of silica exposure which could not be assessed in this study. While this is one of the largest studies assessing the association between silica exposure and SSc, the absolute number of patients with reported exposure to silica remains relatively low (95 patients, 6.6% in this registry). Finally, we did not have gender data and hence it remains to be confirmed whether the worse disease features seen in males with SSc are driven by biological factors (i.e., sex) or occupational/sociocultural exposures (i.e., gender).

Exposure to silica was seen in ∼7% of the CSRG cohort and was more common patients younger at diagnosis, males, smokers and patients with a more severe disease. Differences by sex were observed where male patients exposed to silica were more often smokers and Caucasian vs. silica-exposed female patients were younger at SSc diagnosis. Furthermore, silica exposure was associated with worse SSc outcomes in these patients such as younger age at SSc diagnosis and a strong trend toward higher risk of dcSSc, ATA antibodies, more severe GI disease, ILD, and mortality. Hence, our results suggest that prior silica exposure among SSc patients in North America is common, particularly among males and younger females and these patients are at risk of worse outcomes. Patients with occupational silica exposure who present with new onset Raynaud’s phenomenon should be thoroughly assessed for Very Early Diagnosis Of SSc (VEDOSS) through physical examination (e.g., puffy fingers), nailfold capillaroscopy (using dermoscopy or videocapillaroscopy), and antibody testing (i.e., antinuclear and/or SSc-specific antibodies). Earlier diagnosis of SSc could lead to counseling about discontinuation of silica exposure as well as earlier screening for systemic involvement and prompt treatment initiation. For patients diagnosed with SSc, ILD is considered to be an early complication often occurring in the first 3–5 years of disease onset. While there are no clear guidelines, experts usually suggest baseline ILD screening with high resolution CT scan and PFTs with DLCO and regular monitoring by spirometry for the first 3–5 years. SSc patients with silica exposure could benefit from regular follow up during the first 5 years and beyond. As any SSc patients, clinical signs or features of ILD should prompt an early specialist referral to minimize complications. Unfortunately, there is no specific treatment available for SSc associated with silica exposure aside from discontinuing exposure, smoking cessation and SSc management. Large prospective studies with detailed exposure/occupational questionnaires and job matrices are needed to further study the association between silica exposure and prognosis of patients with SSc.

## Data availability statement

The data analyzed in this study is subject to the following licenses/restrictions: Canadian Scleroderma Research Group has the dataset. Requests to access these datasets should be directed to MB.

## Ethics statement

The studies involving human participants were reviewed and approved by Jewish General Hospital, Montreal, Canada and at all participating CSRG study sites. The patients/participants provided their written informed consent to participate in this study.

## Investigators of the Canadian Scleroderma Research Group

M. Baron, Montreal, Quebec; M. Hudson, Montreal, Quebec; G. Gyger, Montreal, Quebec; J. Pope, London, Ontario; M. Larche, Hamilton, Ontario; N. Khalidi, Hamilton, Ontario; A. Masetto, Sherbrooke, Quebec; E. Sutton, Halifax, Nova Scotia; T.S. Rodriguez Reyna, Mexico City, Mexico; N. Maltez, Ottawa, Ontario; C. Thorne, Newmarket, Ontario; P.R. Fortin, Quebec, Quebec; A. Ikic, Quebec, Quebec; D. Robinson, Winnipeg, Manitoba; N. Jones, Edmonton, Alberta; S. LeClercq, Calgary, Alberta; P. Docherty, Moncton, New Brunswick; D. Smith, Ottawa, Ontario; M. J. Fritzler, Department of Medicine, Cumming School of Medicine, Calgary, Alberta.

## Author contributions

AM, RM, ER, MB, and EN designed the study. AM and LO collected and organized the data. AM prepared the manuscript. RM performed the statistical analyses with input from ER. AL, MC, IL, and MH provided feedback on the design, analyses, and manuscript. EN guided and supervised the study. All authors have read and approved the final version of the manuscript.
